# Plant metabolites targeting mitochondrial dysfunction in cardiovascular diseases: pharmacological mechanisms and combination strategies

**DOI:** 10.3389/fphar.2026.1851598

**Published:** 2026-07-16

**Authors:** Yuyao Wang, Sisi He, Hua Li, Junqi Gao, Taiyi Wang

**Affiliations:** 1 Innovation Research Institute of Chinese Medicine, Shandong University of Traditional Chinese Medicine, Jinan, China; 2 Key Laboratory of Traditional Chinese Medicine Classical Theory, Ministry of Education, Shandong University of Traditional Chinese Medicine, Jinan, China; 3 Shandong Key Laboratory of Innovation and Application Research in Basic Theory of Traditional Chinese Medicine, Shandong University of Traditional Chinese Medicine, Jinan, China; 4 Institute of Acupuncture and Moxibustion, Shandong University of Traditional Chinese Medicine, Jinan, China

**Keywords:** cardiovascular diseases, combination therapy, mitochondrial dysfunction, myocardial injury, natural products, oxidative stress

## Abstract

Cardiovascular diseases (CVDs) are closely associated with mitochondrial dysfunction, including impaired mitochondrial biogenesis, abnormal mitochondrial dynamics, excessive oxidative stress, dysregulated mitophagy, and disorders of energy metabolism. Accumulating evidence indicates that natural products exert significant cardioprotective effects by coordinately regulating multiple mitochondrial pathways, thereby alleviating myocardial injury and slowing disease progression. This review systematically summarises recent advances in natural metabolites and their rational combination strategies for cardiovascular diseases, with a particular focus on mitochondrial regulation. Representative metabolites, including flavonoids, alkaloids, saponins, and polyphenols, regulate key signalling pathways such as SIRT1/PGC-1α, AMPK, Nrf2, PINK1/Parkin, and Drp1/MFN2 to restore mitochondrial homeostasis. Importantly, natural metabolite combinations further demonstrate synergistic and complementary effects through coordinated regulation of distinct mitochondrial pathways. For example, EGCG and Rhein cooperatively alleviate myocardial ischemia/reperfusion injury by simultaneously suppressing oxidative stress and TLR4-mediated inflammatory signalling. Collectively, this review highlights the considerable potential of natural products and their rational combinations as therapeutic strategies for cardiovascular diseases through the modulation of mitochondrial function.

## 1 Introduction

Cardiovascular diseases (CVDs) remain the leading cause of death and disability worldwide. According to data from the World Health Organization (WHO) and other research institutions, CVDs account for approximately one-third of global deaths. In most low- and middle-income countries, the burden of CVDs has been steadily increasing over the past few decades. Between 2025 and 2050, the global prevalence of cardiovascular diseases is expected to rise by 90% (Chong et al., 2024). CVDs represent a major public health challenge, encompassing coronary artery disease, hypertension, cardiomyopathy, heart failure, atherosclerosis, dyslipidaemia, hyperglycaemia, stroke, and transient ischaemic attacks, all of which exhibit complex aetiologies (Roth et al., 2020). At present, the management of cardiovascular diseases primarily relies on pharmacological and surgical approaches, such as aspirin, ACE inhibitors, β-blockers, statins, and antibiotics. While these therapies have enhanced symptom control and improved short-term clinical outcomes, treatment options that directly address the underlying mechanisms particularly mitochondrial dysfunction are still inadequate. In light of the significant threat CVDs pose to public health, as well as the considerable social and economic burdens they create, it is imperative to develop novel drugs and more effective therapeutic strategies.

**FIGURE 1:**
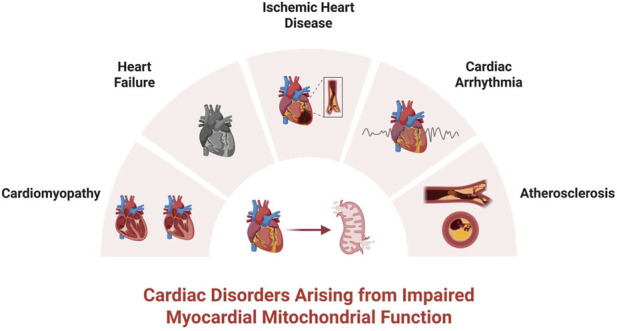
Mitochondrial dysfunction as a central mechanism in cardiovascular diseases.

Mitochondria play a pivotal role in energy production in all multicellular eukaryotes by synthesising ATP and maintaining metabolic homeostasis. Beyond energy metabolism, mitochondria are also involved in regulation of the cell cycle, survival, and apoptosis. Within cardiomyocytes, mitochondria constitute approximately one-third of the total cell volume and are essential for maintaining cardiovascular homeostasis and function. In addition to powering the myocardium via oxidative phosphorylation, they are central to key pathophysiological events, including reactive oxygen species (ROS) signaling, calcium flux, apoptosis, and inflammatory responses (Gao and Hou, 2023). Under stress conditions, myocardial mitochondria struggle to maintain cellular homeostasis and adaptive capacity to meet physiological demands. This leads to reduced ATP synthesis and increased ROS generation, ultimately promoting cardiomyocyte death and impaired cardiac function (Bonora et al., 2019). Accumulating evidence indicates that mitochondrial dysfunction—such as energy metabolism imbalance, excessive oxidative stress, abnormalities in mitochondrial dynamics (fusion/fission imbalance), and defective mitophagy—is closely associated with heart failure, myocardial ischaemia–reperfusion injury, hypertensive cardiomyopathy, and diabetic cardiomyopathy (Zuo et al., 2024). Cardiovascular diseases associated with mitochondrial dysfunction are shown in Figure 1. Accordingly, targeting myocardial mitochondria has been widely validated as a therapeutic strategy for CVDs. However, clinical interventions aimed at mitochondrial dysfunction face substantial challenges, and currently available drugs exhibit notable limitations in membrane permeability and multi-mechanism synergy.

Schematic illustration depicting the pivotal role of myocardial mitochondrial dysfunction in the pathogenesis of major cardiovascular diseases, including ischemic heart disease, cardiac arrhythmia, atherosclerosis, cardiomyopathy, and heart failure.

Natural products, with their multi-target, low-toxicity, and synergistic regulatory properties, offer new perspectives for the prevention and treatment of cardiovascular diseases. Extensive research has confirmed that natural products can inhibit cardiomyocyte apoptosis by regulating mitochondrial autophagy, mitochondrial fusion and fission, improving mitochondrial respiratory function, and reducing ROS production, thereby preserving cardiac cell viability and function (Boeing et al., 2023). Notably, the efficacy of single natural product metabolites is often constrained by low bioavailability or limited target coverage. In contrast, combinations of natural products exert “multi-metabolite, multi-target, and multi-pathway” synergistic effects, enabling a more comprehensive reversal of mitochondrial dysfunction. For example, the combined application of Astragaloside IV and Ginsenoside Rg1 has been shown to alleviate myocardial injury in a hypoxia/reoxygenation model by suppressing excessive mitochondrial autophagy mediated by the HIF-1α/BNIP3 pathway (Xia et al., 2024).

This review integrates current research on the roles of distinct mitochondrial functional modules in CVDs and summarises advances in natural product-based interventions targeting these modules. It proposes a transition from “single-metabolite” approaches to “optimised multi-metabolite formulations” and highlights the potential advantages of multi-metabolite compositions in achieving multi-target synergistic therapy. This framework provides new insights and a theoretical foundation for the prevention and treatment of cardiovascular diseases.

## 2 Methods

To provide a comprehensive overview of natural metabolites targeting mitochondrial dysfunction in cardiovascular diseases (CVDs), a structured literature search was conducted primarily using the PubMed database. Relevant studies published up to January 2026 were systematically screened and summarized.

Initially, literature related to mitochondrial dysfunction in major cardiovascular disorders, including myocardial ischemia/reperfusion injury, myocardial infarction, heart failure, diabetic cardiomyopathy, atherosclerosis, and hypertension, was retrieved to summarize the pathological roles of mitochondrial oxidative stress, mitochondrial dynamics, mitophagy, mitochondrial biogenesis, calcium dysregulation, and energy metabolism disorders in CVD progression.

Subsequently, combinations of keywords related to natural products, mitochondrial function, and cardiovascular protection were used to identify studies investigating the therapeutic potential of natural metabolites. Search terms included “natural products”, “natural metabolites”, “phytochemicals”, “botanical drugs”, “combination strategies”, “drug combinations”, “mitochondria”, “mitochondrial dysfunction”, “mitophagy”, “oxidative stress”, “mitochondrial dynamics”, “energy metabolism”, “cardiovascular disease”, “myocardial ischemia/reperfusion”, “myocardial infarction”, and “diabetic cardiomyopathy”, together with terms such as “treatment”, “therapy”, “improvement”, “protection”, “cardioprotection”, and “amelioration”.

To improve literature coverage and taxonomic accuracy, representative botanical synonyms and alternative taxonomic names were additionally considered where appropriate.

## 3 Cardiovascular diseases and mitochondrial dysfunction

Cardiovascular diseases (CVDs) remain a leading cause of global mortality and are increasingly recognised as disorders closely linked to mitochondrial dysfunction. Beyond ATP production, mitochondria coordinate redox balance, calcium signalling, inflammatory responses, and multiple forms of programmed cell death. Consequently, mitochondrial injury is no longer viewed as a secondary consequence of cardiac stress, but rather as a central driver of myocardial remodelling and functional decline. (Abdullaev et al., 2025; Hinton et al., 2024).

**FIGURE 2:**
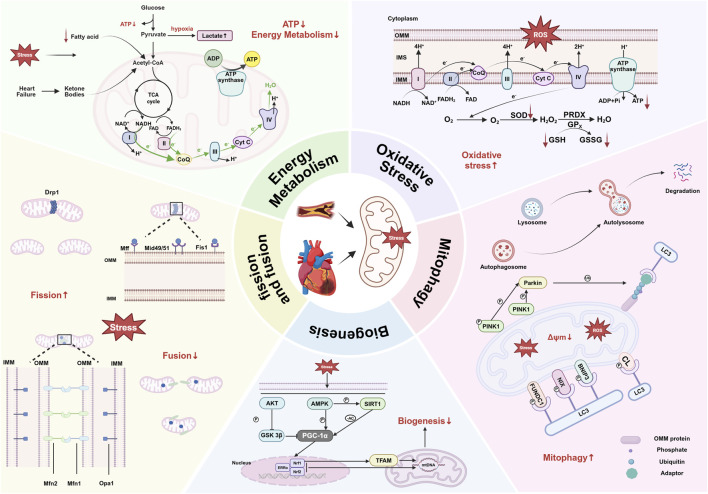
Major mechanisms of mitochondrial dysfunction in cardiovascular diseases. ATP:adenosine triphosphate; Acetyl-CoA: Acetyl coenzyme A; TAC:Tricarboxylic Acid Cycle; NAD+: Nicotinamide adenine dinucleotide; NADH: Reduced form of NAD^+^; FAD:Flavin adenine dinucleotide; FADH2: Reduced form of FAD; CoQ: Coenzyme Q; Cyt C: Cytochrome c. OMM: Outer Mitochondrial Membrane; IMM: Inner Mitochondrial Membrane; Mid49/51: Mitochondrial dynamics proteins of 49/51 kDa; Fis1: Fission 1 protein; Drp1: Dynamin-related protein 1; Mfn2: Mitofusin 2; Mfn1:Mitofusin 1; Opa1: Optic atrophy 1. AKT: Protein Kinase B; p38 MAPK: p38 Mitogen-Activated Protein Kinase; AMPK: AMP-activated protein kinase; SIRT1: Sirtuin 1; GSK 3β: Glycogen Synthase Kinase 3 beta; PGC-1α: Peroxisome proliferator-activated receptor gamma coactivator 1-alpha; ERRα: Estrogen-Related Receptor Alpha; Nrf1: Nuclear Respiratory Factor 1; Nrf2: Nuclear factor erythroid 2-related factor 2; TFAM: Mitochondrial Transcription Factor A. PINK1: PTEN-induced putative kinase 1; Parkin: Parkin RBR E3 Ubiquitin-Protein Ligase; FUNDC1: FUN14 domain-containing protein 1; NIX: BCL2/adenovirus E1B 19-kDa interacting protein 3-like; BNIP3: BCL2/adenovirus E1B 19-kDa interacting protein 3; CL: Cardiolipin; LC3: Microtubule-associated protein 1A/1B-light chain 3; IMS: Intermembrane Space; SOD: Superoxide Dismutase; H2O2: Hydrogen Peroxide; PRDX: Peroxiredoxin; GPX: Glutathione Peroxidase; GSH: Reduced Glutathione; GSSG: Oxidized Glutathione.

In myocardial ischaemia/reperfusion injury, heart failure, diabetic cardiomyopathy, and atherosclerosis, impaired mitochondrial function commonly manifests as reduced oxidative phosphorylation efficiency, excessive ROS accumulation, mitochondrial membrane depolarisation, and mitochondrial DNA damage. ((Poznyak et al., 2020). These abnormalities are highly interconnected rather than independent pathological events. Excessive mitochondrial ROS production can disrupt mitochondrial dynamics, trigger mitophagy, and impair energy metabolism, whereas abnormal mitochondrial fission and fusion may further aggravate oxidative stress and mitochondrial DNA damage. Likewise, mitophagy is not merely a quality control process for removing damaged mitochondria, but also closely linked to mitochondrial biogenesis and metabolic adaptation in maintaining mitochondrial homeostasis. These various dysfunctions exacerbate one another, creating a self-amplifying cycle that accelerates cardiomyocyte injury and disease progression. (Manolis et al., 2021). The pathological mechanisms of various mitochondrial dysfunctions in cardiovascular diseases are illustrated in Figure 2.

Given the limited regenerative capacity and high energetic demand of cardiomyocytes, restoration of mitochondrial homeostasis has emerged as an important therapeutic strategy for CVDs. Current evidence suggests that modulation of mitochondrial biogenesis, mitochondrial dynamics, mitophagy, oxidative stress, and energy metabolism may collectively improve cardiac resilience. Importantly, natural metabolites often regulate several mitochondrial pathways simultaneously rather than acting through a single molecular target.

### 3.1 Mitochondrial biogenesis and CVD

Mitochondrial biogenesis is essential for maintaining myocardial energy supply and adapting to metabolic stress. The PGC-1α/NRF1/TFAM axis represents the core regulatory network governing mitochondrial DNA replication, respiratory chain assembly, and oxidative phosphorylation. (Li et al., 2021a; Rubio-Tomás et al., 2024). In failing or stressed myocardium, suppression of PGC-1α signalling is frequently associated with reduced mitochondrial content, impaired ATP production, and progressive contractile dysfunction. (Naumenko et al., 2022; Rabinovich-Nikitin et al., 2022).

AMPK and SIRT1 act as major upstream energy sensors that regulate PGC-1α activity through phosphorylation and deacetylation, respectively. Although activation of this pathway is generally considered cardioprotective, current evidence also suggests that excessive or uncontrolled stimulation of mitochondrial biogenesis may not always be beneficial, particularly under conditions of severe oxidative stress where newly generated mitochondria may remain dysfunctional. Therefore, restoration of mitochondrial quality rather than simple expansion of mitochondrial quantity is likely to be more clinically relevant.

In addition, PGC-1α cooperates closely with PPARγ to coordinate fatty acid utilisation, antioxidant responses, and mitochondrial turnover. (Chambers and Wingert, 2020; Xie et al., 2024). Dysregulation of this network contributes to metabolic inflexibility and lipid accumulation in cardiomyocytes, which are characteristic features of heart failure and diabetic cardiomyopathy. Collectively, these findings highlight mitochondrial biogenesis as an adaptive but tightly regulated process in CVD progression.

### 3.2 Mitochondrial fusion and fission in CVD

#### 3.2.1 Mitochondrial fusion and CVD

Mitochondrial fusion and fission are dynamic and interdependent processes that determine mitochondrial morphology, quality control, and metabolic adaptability. In cardiomyocytes, disruption of this balance contributes directly to oxidative stress, calcium dysregulation, and cell death. (Peñalva et al., 2024). Mitochondrial fusion is primarily mediated by MFN1/2 and OPA1, which maintain mitochondrial connectivity and cristae integrity. Reduced MFN2 or OPA1 expression has been linked to impaired oxidative phosphorylation, excessive ROS production, and increased susceptibility to cardiomyocyte apoptosis in conditions such as doxorubicin-induced cardiomyopathy and myocardial ischaemia/reperfusion injury. (Ding et al., 2022a; Huang et al., 2024). Notably, MFN2 also regulates mitochondria–ER interactions, suggesting that defective fusion affects not only mitochondrial morphology but also calcium signalling and metabolic homeostasis.

#### 3.2.2 Mitochondrial fission and CVD

Conversely, mitochondrial fission is mainly controlled by Drp1. Under pathological stress, excessive Drp1 activation promotes mitochondrial fragmentation, inflammatory activation, and energetic collapse. (Chen et al., 2022; Rios et al., 2023). Increased Drp1 activity has been implicated in myocardial infarction, atherosclerosis, and heart failure, where abnormal mitochondrial fragmentation amplifies ROS production and NLRP3 inflammasome activation. (Su et al., 2023).

Although modulation of mitochondrial dynamics is widely regarded as a promising therapeutic strategy, current studies remain largely preclinical, and the precise threshold between adaptive and maladaptive mitochondrial remodelling is still incompletely understood. This limitation partly explains why translation of mitochondrial dynamics-targeted therapies into clinical practice remains challenging.

### 3.3 Mitochondrial autophagy and CVD

Mitophagy is a selective quality-control mechanism responsible for eliminating dysfunctional mitochondria and preserving mitochondrial homeostasis. (Pan et al., 2025). In the cardiovascular system, insufficient mitophagy leads to accumulation of damaged mitochondria, whereas excessive mitophagy may contribute to mitochondrial depletion and energetic failure. Thus, mitophagy appears to exert a context-dependent role during CVD progression rather than functioning as a uniformly protective process.

The PINK1/Parkin pathway is the best-characterised ubiquitin-dependent mitophagy mechanism. Under mitochondrial stress, accumulation of PINK1 on the outer mitochondrial membrane recruits and activates Parkin, promoting ubiquitination of mitochondrial proteins and subsequent autophagic clearance (Narendra and Youle, 2024; Zhao et al., 2024). Impairment of this pathway has been associated with ROS accumulation, inflammasome activation, and cardiomyocyte apoptosis during diabetes and myocardial ischaemia/reperfusion injury. (Yang N. et al., 2024).

In parallel, receptor-mediated pathways involving BNIP3/NIX and FUNDC1 directly interact with LC3 proteins to initiate mitophagy under hypoxic or metabolic stress (Sulkshane et al., 2021). However, sustained activation of BNIP3-dependent mitophagy may aggravate mitochondrial depletion and contractile dysfunction in failing hearts, indicating that excessive mi.

Lipid-mediated mitophagy, particularly cardiolipin-dependent signalling, further links mitochondrial membrane integrity to autophagic regulation. (Iriondo et al., 2022). Altered cardiolipin remodelling has been implicated in ageing-related cardiovascular disorders, obesity-associated cardiomyopathy, and atherosclerosis, highlighting the broader metabolic relevance of mitophagy dysfunction.

Overall, current evidence suggests that therapeutic modulation of mitophagy requires a careful balance between mitochondrial clearance and mitochondrial preservation. Simply enhancing autophagy may not necessarily improve cardiac outcomes unless mitochondrial quality and metabolic recovery are simultaneously restored.

### 3.4 Mitochondrial oxidative stress and CVD

Mitochondrial oxidative stress is a central pathological feature of many CVDs. During oxidative phosphorylation, mitochondria continuously generate low levels of ROS, which normally function as signalling molecules and are efficiently neutralised by endogenous antioxidant systems. (Rogov et al., 2021). Under pathological conditions, however, excessive ROS production overwhelms antioxidant capacity, leading to mitochondrial DNA damage, respiratory chain dysfunction, and progressive impairment of ATP synthesis. (Senoner and Dichtl, 2019).

In myocardial ischaemia/reperfusion injury, abrupt restoration of oxygen supply markedly increases mitochondrial ROS generation, contributing to calcium overload, mitochondrial permeability transition, and cardiomyocyte death. (Andreadou et al., 2021). Persistent oxidative stress also amplifies inflammatory signalling, promotes endothelial dysfunction, and accelerates ventricular remodelling and fibrosis. (Liu Y. et al., 2021). Importantly, oxidative stress rarely acts alone. It interacts closely with inflammation, ferroptosis, ER stress, and metabolic dysfunction, collectively driving disease progression. (Wang et al., 2025).

This interconnected nature may explain why antioxidant therapies targeting single ROS sources have shown limited clinical success despite promising experimental findings. Consequently, current research is increasingly shifting from simple ROS scavenging toward integrated restoration of mitochondrial redox homeostasis and metabolic function.

### 3.5 Mitochondrial energy metabolism and CVD

Cardiomyocytes rely heavily on mitochondrial oxidative phosphorylation to sustain continuous contractile activity. Under physiological conditions, fatty acid oxidation provides most myocardial ATP, whereas glucose and ketone metabolism serve complementary roles. (Liu et al., 2025).

During myocardial ischaemia or heart failure, impaired mitochondrial respiration forces cardiomyocytes to shift toward glycolytic metabolism. Although this metabolic adaptation temporarily supports survival, prolonged reliance on glycolysis is energetically inefficient and contributes to ATP depletion, contractile dysfunction, and adverse remodelling. (Ranjbarvaziri et al., 2021). In parallel, impaired fatty acid utilisation promotes accumulation of toxic lipid intermediates, which further exacerbate oxidative stress, ER stress, and cardiomyocyte apoptosis (Ajoolabady et al., 2024). Key metabolic regulators, including AMPK, SIRT1, PGC-1α, and PPARs, coordinate substrate utilisation and mitochondrial adaptability (Sun et al., 2023). Disruption of these pathways impairs metabolic flexibility and accelerates progression toward heart failure. In addition, abnormalities in mitochondrial, ETC., components, coenzyme Q10 availability, and tricarboxylic acid cycle activity further compromise ATP production (Bulló et al., 2021; Gutierrez-Mariscal et al., 2021; Kumar et al., 2022).

Notably, growing evidence suggests that metabolic dysfunction in CVD is not simply a consequence of mitochondrial injury but may itself actively reshape inflammatory and cell death pathways. Therefore, therapeutic strategies aimed at restoring mitochondrial metabolism are increasingly viewed as a means to simultaneously modulate oxidative stress, inflammation, and myocardial survival rather than merely improving ATP production alone.

## 4 Natural products and combinations in mitochondrial pathways for CVD treatment

**FIGURE 3:**
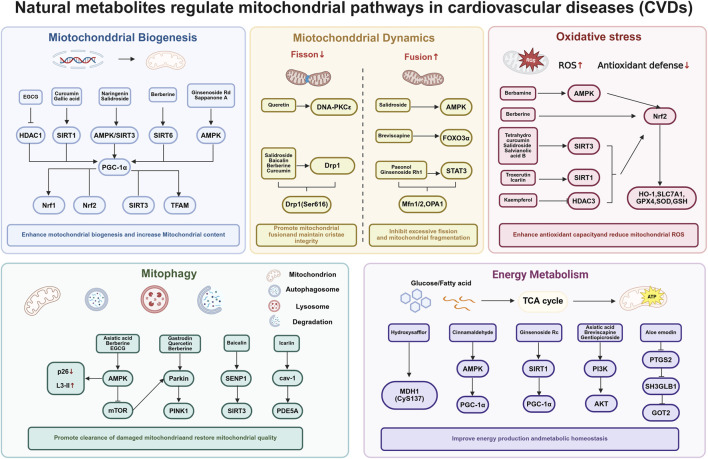
Mechanisms of natural products and their metabolites related to mitochondrial dysfunction in cardiovascular diseases.

The process by which natural products ameliorate cardiovascular diseases via mitochondrial pathways and related pathways is shown in Table 1 and Figure 3.

**TABLE 1:** Effects of natural products on mitochondrial function in CVD.

Mitochondrial function	Metabolite name	Metabolite type	Animal or cell models	Dosage	Pathway	References
Mitochondrial Biogenesis	curcumin	Diarylheptanoid	SCM(C57BL/6:LPS HL-1:LPS)	Animal: 80 mg/kg Cell: 20 μM	SIRT1-DRP1/PGC-1α	Hou et al. (2024)
	Ginsenoside Rd	Terpenoid - Saponin	HF(C57BL/6: TAC CAL,ISO,LPS; 3T3-L1,H9c2,NRVMs: OGD)	Animal: 20 mg/kg Cell: 20 μM	TBK1-AMPK, WNT5A/Ca^2+^	Wan et al. (2023)
	Gallic acid	Phenolic Acid	MI(Wistar:ISO)	Animal: 0.5,1,2 mL/kg	SIRT1-PGC-1α-Nrf2-TFAM	Zarei et al. (2023)
	Naringenin	Flavonoid	I/R (SD: LAD; H9c2:SIR)	Animal: 50 mg/kg Cell: 80 μM	AMPK–PGC-1α–SIRT3	Yu et al. (2019)
	Epigallocatechin-3-gallate	Flavonoid	Cardiac hypertrophy (SD:TAC, ; H9c2,NRVMs:PE)	Animal: 100 mg/kg Cell: 10 μM	HDAC1-NRF1/PGC-1α	Li et al. (2024)
	Sappanone A	homoisoflavonoid	I/R (Wistar: LAD)	100 μM	AMPK-PGC-1α	Shi et al. (2021)
	Salidroside	Phenylethanoid	DCM(C57BLKS/J:HFD+50mg/kgSTZ; H9c2:high glucose)	Animal: 100 mg/kg Cell: 10 μM	AMPK–PGC-1α–TFAM	Li et al. (2021b)
	Berberine	Alkaloid	Cardiac remodeling (C57BL/6J: HFD) CIH(C57BL/6J:OSA)	Animal: 200 mg/kg 30 mg/kg	SIRT6-AMPK-FOXO3a; KLF4	Ding et al. (2021), Zhou et al. (2024)
Fusion and fisson	Salidroside	Phenylethanoid	I/R (SD:LAD; H9c2:H/R)	Animal: mg/kg Cell: 10 μM	AMPK–ERS–Mfn2/Opa1; Drp1	Tian (2022)
	Berberine	Alkaloid	HFpEF(C57BL/6N:HFD+L-NAME)	Animal: 50 mg/kg	Drp1-Ca^2+^	Abudureyimu et al. (2023)
	Baicalin	Flavonoid	I/R (SD:CA; H9c2:H/R)	Animal: 100 mg/kg Cell: 20 μM	Drp1(Ser616)	Wu et al. (2021)
	Paeonol	Phenol/Monoterpenoid	DCM(SD:STZ; NRCMs: high glucose); DIC(SD:dox; NRCMs:DOX)	Animal: 300 mg/kg Cell: 100 μM,50 μM	CK2α–Jak2–Stat3–Opa1 PKCε–Stat3–Mfn2	Liu C. et al. (2021), Ding et al. (2022b)
	Breviscapine	Flavonoid	HF(C57BL/6: TAC ;NRCMs, NRCFs,H9c2:PE)	Animal: 50 mg/kg Cell:10–50 μg/mL	FOXO3a-MFN-1	Lin et al. (2024)
	Ginsenoside Rh1	Terpenoid - Saponin	MI(C57BL/6:LAD; H9c2,NRVMs:OGD)	Animal: 2.5,5,10 mg/kg Cell: 1,5,25 μM	SIRT3/Foxo3a	Gong et al. (2025)
	Quercetin	Flavonoid	I/R (C57BL/6:LAD,SIRT5f/f,SIRT5CKO,SIRT5TG-CMs:H/R)	Animal: 10,30,50 mg/kg Cell: 166,332,500 μM	DNA-PKcs,Fis1, DRP1	Chang et al. (2024)
	Curcumin	Diarylheptanoid	SCM(C57BL/6:LPS HL-1:LPS)	Animal: 80 mg/kg Cell: 20 μM	SIRT1- DRP1	Hou et al. (2024)
Mitophagy	Baicalin	Flavonoid	DCM(db/db:HFD H9c2:high glucose)	Animal: 100 mg/kg Cell: 30 μM	SENP1/SIRT3,deSUMOylation	Zhang P. et al. (2025)
	Berberine	Alkaloid	I/R (C57BL/6:LAD,H9c2:H/R); HF(C57BL/6J:TAC; NMCMs:PE)	Animal: 50 mg/kg Cell: 5 μM	RhoE/AMPK; PINK1/Parkin	Hu et al. (2024), Abudureyimu et al. (2020)
	Gastrodin	Phenolic Glycoside	I/R (C57BL/6:LAD,H9c2:H/R)	Animal: 150 mg/kg Cell: 10 μM	PINK1/Parkin	Chen et al. (2024)
	Epigallocatechin Gallate	Flavonoid	DIC(C57BL/6:DOX,H9c2,NRCMs:DOX)	Animal: 20 mg/kg Cell: 20 μM	AMPKα2	He et al. (2021)
	Icariin	Flavonoid	DIC(H9c2:DOX)	Cell: 1.5 μM	Caveolin-1,PDE5a	Scicchitano et al. (2021)
	Quercetin	Flavonoid	I/R (C57BL/6:LAD,SIRT5f/f,SIRT5CKO,SIRT5TG-CMs:H/R)	Animal: 10,30,50 mg/kg Cell: 166,332,500 μM	DNA-PKcs,SIRT5	Chang et al. (2024)
Oxidative stress	Berberine	Alkaloid	DIC(SD:DOX,NRCMs,CFs:DOX); DCM(db/db,H9c2:high glucose)	Animal: 60 mg/kg 136.5 mg/kg Cell: 1 μM; 20 μM	Nrf2/HO-1/TFAM; mTOR/mtROS	Wang et al. (2023), Zhong et al. (2024)
	Troxerutin	Flavonoid	DIC(Wistar:DOX)	Animal: 150 mg/kg	SIRT-1/PGC-1α/NRF-2	Babaei-Kouchaki et al. (2020)
	Berbamin	Alkaloid	I/R (C57BL/6:LAD)	Animal: 20 mg/kg	AMPK/Nrf2	Xu et al. (2022)
	Kaempferol	Flavonoid	I/R (SD:ISO,H9c2:CoCl_2_); DIC (H9c2,AC16:DOX)	Animal: 20 mg/kg Cell: 20 μM; 40 80,160	HDAC3-Nrf2 NRF2/SLC7A11/GPX4	Yue et al. (2024), Zhang L. et al. (2025)
	Tetrahydrocurcumin	Diarylheptanoid	HF post-MI(C57BL/6:LAD,NCMs:Hypoxia)	Animal: 120 mg/kg Cell: 5 μM	Nrf2/SIRT3	Zhang et al. (2023)
	Icariin	Flavonoid	ICR(C57BL/6,H9c2:40 μM Cisplatin)	Animal: 30 mg/kg Cell: 12 μM	PI3K/Akt,SIRT1/MAPKs	Xia et al. (2022)
	Salvianolic acid B	Phenolic Acid	I/R (SD:LAD,H9c2:H/R)	Animal: 20 mg/kg Cell: 5 μM	SIRT3	Wei et al. (2025)
	Ginsenoside Rb1	Terpenoid - Saponin	I/R (C57BL/6:LAD,AMCMs,H9c2:H/R)	Animal: 50 mg/kg Cell: 10 μM	ND3	Jiang et al. (2021)
	Salidroside	Phenylethanoid	I/R (SD:LAD,H9c2:H/R)	Animal: 50 mg/kg Cell: 10 μM	AMPKα2	Yang B. et al. (2024)
Energy metabolism	Ginsenoside rc	Terpenoid - Saponin	I/R (SD:LAD,CCA)	Animal:10 mg/kg	SIRT1-PGC1α	Huang et al. (2021)
	Aloe emodin	Alkaloid - Anthraquinone	RIHD (SD:X-ray; zebrafish: L-kynurenine)	Animal:10 mg/kg zebrafish:1 μM	PTGS2/SH3GLB1/NDP52; GOT2	Ouyang et al. (2024)
	Asiatic acid	Terpenoid - Triterpenoid	MI(C57BL/6:LAD,NRCMs:OGD)	Animal: 25 mg/kg Cell: 10 μM	PI3K/Akt	Qiu et al. (2022)
	Breviscapine	Flavonoid	DIC(C57BL/6:DOX,H9c2:DOX)	Animal: 8.16 mg/kg Cell: 200 μM	5-HT-PI3K/Akt	Li et al. (2022)
	Cinnamaldehyde	Aromatic Aldehyde	HF(SD:ISO,NRCMs:ISO)	Animal: 80 mg/kg Cell: 40 μM	GRK2-AMPK/PGC-1α	Xu et al. (2024)
	Gentiopicroside	Iridoid Glycoside	T2DM (C57BL/6:STZ+HFD; C2C12:PA)	Animal: 25.50.100 mg/kg Cell: 50–100 μM	DDB2-PAQR3-p110α-PI3K/AKT	Xiao et al. (2022)
	Hydroxysafflor yellow A	Flavonoid	MIRI(C57BL/6:LAD; H9c2:H/R)	Animal: 25 40.80 mg/kg Cell: 50,100 200 μM	MDH1-Cys137	Zhao et al. (2025)

Molecular targets and signaling pathways modulated by diverse natural products across key mitochondrial phenotypes in cardiovascular disease, including bioenergetics, redox balance, dynamics, and quality control.

### 4.1 Natural products and mitochondrial biogenesis in CVD

In recent years, numerous natural products have been shown to regulate mitochondrial biogenesis–related signalling pathways and to play significant roles in cardiac protection. These natural products primarily promote mitochondrial biogenesis by modulating the sirtuin family, the AMPK signalling axis, and key transcription factors.

Curcumin, a diarylheptanoid metabolite derived from *Curcuma longa* L. (Zingiberaceae), activates SIRT1 and upregulates the expression of PGC-1α, TFAM, and Nrf2, while reducing Drp1 translocation from the cytoplasm to mitochondria, thereby improving mitochondrial function in cardiomyocytes. However, these protective effects were largely abolished in SIRT1-knockout HL-1 cells following LPS stimulation, suggesting that the mitochondrial protective actions of curcumin are highly dependent on SIRT1 signalling (Hou et al., 2024). Ginsenoside Rd, a triterpenoid saponin metabolite isolated from *Panax ginseng* C.A.Mey. (Araliaceae), promotes mitochondrial biogenesis via the TBK1–AMPK pathway by increasing omentin secretion in adipocytes and through activation of the Wnt5a/Ca^2+^ signalling pathway to improve mitochondrial function and reduce myocardial ischaemic injury (Wan et al., 2023). Gallic acid, a phenolic acid metabolite widely distributed in multiple botanical drugs, including *Paeonia lactiflora* Pall. (Paeoniaceae), regulates the expression of SIRT1, PGC-1α, Nrf2, and TFAM to promote mitochondrial biogenesis in the myocardium, thereby alleviating aging-associated cardiac hypertrophy in rats (Zarei et al., 2023). Naringenin, a flavanone metabolite isolated from *Citrus × aurantium* L. (Rutaceae), protects mitochondrial biogenesis by inhibiting mitochondrial oxidative stress through the AMPK–SIRT3 signalling pathway, alleviating myocardial ischaemia/reperfusion injury (Yu et al., 2019). Epigallocatechin-3-gallate (EGCG) activates PGC-1α transcription by inhibiting HDAC1, leading to histone H3K9 and H3K14 acetylation at the PGC-1α promoter region. This upregulates NRF1 expression and enhances TFAM and FUNDC1 expression, thereby coordinating mitochondrial biogenesis and mitophagy to attenuate compensatory cardiac hypertrophy (Li et al., 2024).

AMPK knockdown experiments further demonstrated that Sappanone A, a homoisoflavanone metabolite isolated from *Caesalpinia sappan* L. (Fabaceae), post-conditioning increases mitochondrial DNA replication and PGC-1α expression in an AMPK-dependent manner, thereby promoting mitochondrial biogenesis. It also balances mitochondrial dynamics by regulating fusion and enhancing mitophagy, ultimately alleviating myocardial ischemia/reperfusion injury in rats (Shi et al., 2021). Salidroside protects mitochondrial function by activating AMPK, downregulating the expression of TFAM and PGC-1α, and facilitating the translocation of SIRT3 from the cytoplasm into the mitochondria. This process subsequently drives MnSOD deacetylation, and improves diabetic cardiomyopathy in mice (Li et al., 2021b). Berberine, an isoquinoline alkaloid metabolite isolated from *Coptis chinensis* Franch. (Ranunculaceae), through the KLF4 signalling pathway, improves diet-induced pathological cardiac remodelling and mitochondrial dysfunction (Ding et al., 2021). In addition, By stimulating the SIRT6–AMPK–FOXO3a signaling cascade, berberine facilitates both mitochondrial biogenesis and PINK1–Parkin–dependent mitophagy, thereby attenuating myocardial damage elicited by chronic intermittent hypoxia (Zhou et al., 2024).

Notably, although these metabolites all promote mitochondrial biogenesis, their mechanisms differ substantially. Curcumin, gallic acid, and troxerutin mainly regulate the SIRT1/PGC-1α axis and are closely associated with antioxidant defence, whereas ginsenoside Rd and sappanone A exhibit stronger effects on mitochondrial quality control by coordinating mitochondrial biogenesis. In contrast, EGCG exerts broader upstream transcriptional regulation through HDAC1 inhibition-mediated activation of PGC-1α. These findings suggest that natural metabolites may target distinct stages of mitochondrial biogenesis under different pathological conditions.

Although these natural metabolites exert cardioprotective effects through distinct mechanisms regulating mitochondrial biogenesis, most current studies rely predominantly on gene knockdown approaches at the cellular level. Validation using genetically engineered animal models remains limited, and further pharmacological and translational investigations are still required to strengthen the mechanistic evidence and therapeutic relevance of these findings.

### 4.2 Natural products and mitochondrial fusion and fission in CVD

In addition to regulating mitochondrial biogenesis, maintaining the balance of mitochondrial dynamics (fusion and fission) is critical for cardiomyocyte homeostasis. Several natural products have been shown to modulate key proteins involved in mitochondrial fusion and fission, thereby exerting protective effects in cardiovascular disease models.

By inhibiting mitochondrial fission, salidroside blocks Drp1 translocation from the cytoplasm to mitochondria both *in vitro* and *in vivo*. Pharmacological inhibition and gene-silencing experiments further confirmed that this effect is mediated through activation of the AMPK signalling pathway, which reduces endoplasmic reticulum stress and prevents mitochondrial fission and apoptosis, thereby protecting against myocardial ischaemia/reperfusion injury (Tian, 2022). Similarly, berberine inhibits Drp1 mitochondrial localisation, suppresses mitochondrial fragmentation, and prevents intracellular calcium overload. Furthermore, it facilitates autophagic flux and boosts calcium sequestration into the sarcoplasmic reticulum, thereby alleviating the diastolic dysfunction observed in models of heart failure with preserved ejection fraction (HFpEF) (Abudureyimu et al., 2023). In a cardiac arrest–induced myocardial injury model, baicalin, a flavonoid metabolite isolated from *Scutellaria baicalensis* Georgi (Lamiaceae), protects the ischaemic myocardium by inhibiting Drp1-mediated excessive mitochondrial fission, Drp1 overexpression abolished the cardioprotective effects of baicalin, whereas pharmacological inhibition of Drp1 largely mimicked its actions, suggesting its potential as a therapeutic agent for post-cardiac arrest myocardial injury (Wu et al., 2021).

Conversely, several natural metabolites exert cardioprotective effects by promoting mitochondrial fusion. Paeonol, a phenolic metabolite derived from *Paeonia suffruticosa* Andrews (Paeoniaceae), has been identified as a novel activator of mitochondrial fusion, initiating MFN2-mediated mitochondrial fusion through activation of the STAT3 transcription factor via PKCε–STAT3 interaction (Ding et al., 2022b). Paeonol also interacts with CK2α to restore its kinase activity, thereby promoting JAK2–STAT3 phosphorylation and promoting OPA1 transcription, which protects against hyperglycaemia-induced oxidative mitochondrial injury and diabetic cardiomyopathy (Liu C. et al., 2021). In addition, breviscapine, a flavonoid-rich metabolite mixture derived from *Erigeron breviscapus (Vaniot) Hand.-Mazz*. (Asteraceae), promotes nuclear translocation of FOXO3a, which in turn enhances MFN1-mediated mitochondrial fusion, reduces mitochondrial ROS production, and provides a potential therapeutic approach for heart failure (Lin et al., 2024). However, the inhibition of fission or promotion of fusion is not absolute. Ginsenoside Rh1, a triterpenoid saponin metabolite isolated from *P. ginseng* C.A.Mey. (Araliaceae), can modulate mitochondrial dynamics in myocardial ischemia by either activating MFN2 and OPA1 or suppressing Drp1 phosphorylation at Ser616, depending on the mitochondrial status, thereby restoring mitochondrial dynamic homeostasis, thereby alleviating ischemic myocardial injury (Gong et al., 2025).

In summary, these natural metabolites exert cardioprotective effects through precise regulation of mitochondrial fission and fusion. For example, salidroside, berberine, and baicalin primarily inhibit excessive Drp1-mediated mitochondrial fission under acute stress conditions, whereas paeonol and breviscapine mainly promote mitochondrial fusion through MFN1/2- and OPA1-related pathways However, most current mechanistic studies rely predominantly on H9c2 rat cardiomyoblasts or primary rodent cardiomyocytes, which may not fully recapitulate the characteristics of injured human cardiomyocytes. The application of induced pluripotent stem cell-derived cardiomyocytes (iPSC-CMs) may provide more clinically relevant models for future translational studies.

### 4.3 Natural products and mitochondrial autophagy in CVD

Mitochondrial autophagy is a key mechanism for the selective clearance of damaged or dysfunctional mitochondria and plays a pivotal role in maintaining cardiomyocyte health. Numerous natural products have been shown to activate or regulate mitophagy pathways and effectively alleviate various forms of cardiovascular injury.

Baicalin enhances mitophagy and mitochondrial stability by promoting SIRT3 deSUMOylation via the SENP1 pathway, thereby preventing cardiomyocyte death and improving diabetic cardiomyopathy (Zhang P. et al., 2025). Berberine protects mitochondrial function by suppressing excessive autophagy via the RhoE/AMPK signalling pathway, thereby preventing autophagic cell death, restoring energy supply, and maintaining redox homeostasis in cardiomyocytes subjected to ischemia/reperfusion injury (Hu et al., 2024). Gastrodin, a phenolic glycoside metabolite isolated from *Gastrodia elata* Blume (Orchidaceae), promotes mitophagy by activating the PINK1/Parkin pathway, thereby protecting the heart through the removal of damaged mitochondria, Importantly, its therapeutic efficacy was consistently validated in H/R-induced H9c2 cells, ischemia/reperfusion mouse models, and patients undergoing ischemic cardiac surgery (Chen et al., 2024). Epigallocatechin gallate, a catechin metabolite isolated from *Camellia sinensis* (L.) Kuntze (Theaceae), (EGCG) upregulates AMPKα2, enhances adaptive autophagy and mitochondrial function, and modulates miR-30a expression via exosomes to alleviate acute myocardial infarction by regulating apoptosis and autophagy (He et al., 2021; Li et al., 2024). Icariin, a prenylated flavonoid metabolite isolated from *Epimedium brevicornu* Maxim. (Berberidaceae), restores physiological autophagy levels by regulating Cav-1 expression and modulating PDE5a activity, thereby protecting cardiomyocytes against doxorubicin-induced cardiotoxicity (Scicchitano et al., 2021). Quercetin, a flavonol metabolite widely distributed in multiple botanical drugs, including *Sophora japonica* L. (Fabaceae), stabilises DNA-PKcs through SIRT5-mediated desuccinylation, coordinating mitochondrial quality control and metabolic homeostasis in cardiomyocytes and providing a multi-target strategy for cardiovascular protection (Chang et al., 2024).

Collectively, these natural metabolites exert cardioprotective effects through diverse mechanisms involving activation or suppression of autophagy, regulation of post-translational modifications of key proteins, and modulation of pathways such as PINK1/Parkin signalling. These findings provide mechanistic support for the treatment of diabetic cardiomyopathy, myocardial ischemia/reperfusion injury, and related cardiovascular disorders. Interestingly, berberine suppresses excessive autophagy through AMPK signalling, whereas EGCG promotes adaptive autophagy via AMPK activation. This apparent divergence not only highlights the dual nature of autophagy in cardiovascular pathology, but also suggests that AMPK signalling itself may exert context-dependent bidirectional regulatory effects.

### 4.4 Natural products and mitochondrial oxidative stress in CVD

Mitochondria, as the primary source of intracellular ROS, play a central role in myocardial injury induced by oxidative stress. A variety of natural products exhibit marked protective effects against mitochondrial oxidative stress, primarily through activation of endogenous antioxidant signalling pathways, regulation of mitochondrial function, and suppression of inflammation.

Berberine activates the Nrf2 pathway, inhibits mitochondrial dysfunction and oxidative stress, and thereby attenuates doxorubicin-induced myocardial injury and fibrosis (Wang et al., 2023). In addition, berberine inhibits mTOR phosphorylation, reduces mitochondrial ROS production and suppresses NLRP3 inflammasome activation, reduces heat shock protein accumulation, and improves cardiac injury in diabetic cardiomyopathy (Zhong et al., 2024). Troxerutin preconditioning restores the SIRT1/PGC-1α/NRF2 signalling network in the myocardium, enhances antioxidant enzyme activity, and reduces mitochondrial ROS generation to protect against doxorubicin-induced cardiotoxicity (Babaei-Kouchaki et al., 2020). Berbamine, a bisbenzylisoquinoline alkaloid metabolite derived from *Berberis amurensis* Rupr. (Berberidaceae), activates AMPK, promotes Nrf2 nuclear translocation, and upregulates NQO1 and HO-1 expression, thereby reducing oxidative stress and cardiomyocyte apoptosis in myocardial ischaemia/reperfusion injury (Xu et al., 2022).

Kaempferol, a flavonol metabolite widely distributed in multiple botanical drugs, including *Kaempferia galanga* L. (Zingiberaceae), promotes Nrf2 nuclear translocation through suppression of HDAC3 expression, thereby activating downstream antioxidant pathways and attenuating oxidative stress (Yue et al., 2024), Kaempferol activates the NRF2/SLC7A11/GPX4 axis to regulate iron homeostasis and lipid metabolism, thereby inhibiting ferroptosis and alleviating doxorubicin-induced myocardial injury (Zhang L. et al., 2025). Similarly, Tetrahydrocurcumin, a hydrogenated curcumin metabolite derived from *Curcuma longa* L. (Zingiberaceae), enhances SIRT3 expression through activation of Nrf2 signalling, while SIRT3 subsequently deacetylates SOD2 and FOXO3a, reduces oxidative stress, and preserves mitochondrial function (Zhang et al., 2023). Icariin protects cardiomyocytes from oxidative stress–induced apoptosis by activating SIRT1 expression and inhibiting ROS-mediated MAPK signalling pathways (Xia et al., 2022). Salvianolic acid B, a polyphenolic metabolite derived from *Salvia miltiorrhiza* Bunge (Lamiaceae), interacts directly with and upregulates SIRT3, thereby attenuating the expression of Ac-MnSOD and key pyroptotic markers, including NLRP3, ASC, caspase-1, and GSDMD. Through coordinated inhibition of oxidative stress and inflammatory responses, salvianolic acid B effectively attenuates myocardial infarction injury (Wei et al., 2025). Ginsenoside Rb1, a triterpenoid saponin metabolite isolated from *Panax ginseng* C.A.Mey. (Araliaceae), may bind specifically to the ND3 subunit of mitochondrial complex I, thereby selectively inhibiting its NADH dehydrogenase activity and modulating conformational transitions, which effectively attenuates the burst of reactive oxygen species (ROS) during early reperfusion and subsequent myocardial injury (Jiang et al., 2021). Under conditions of mild mitochondrial ROS production, salidroside, a phenylpropanoid glycoside metabolite isolated from *Rhodiola rosea* L. (Crassulaceae), pretreatment upregulates and promotes phosphorylation of AMPKα2, leading to enhanced activity of mitochondrial complex I, Induction of adaptive survival pathways and enhanced cellular resilience against hypoxia/reoxygenation (H/R) insults (Yang B. et al., 2024).

Current evidence demonstrates that numerous natural metabolites exert significant cardioprotective effects through the regulation of mitochondrial oxidative stress. Beyond acting as simple free radical scavengers, most of these metabolites achieve therapeutic effects by activating endogenous antioxidant defence systems and restoring mitochondrial function. Among the identified pathways, Nrf2 signalling appears to represent a common regulatory target of many natural metabolites. For example, both kaempferol and tetrahydrocurcumin activate downstream antioxidant responses through Nrf2-dependent mechanisms. In contrast, troxerutin, a flavonoid metabolite derived from *Sophora japonica* L. (Fabaceae), restores mitochondrial biogenesis through modulation of the SIRT1/PGC-1α pathway, thereby reducing ROS leakage at its source. Collectively, these findings not only highlight oxidative stress as a critical therapeutic target in cardiovascular diseases, but also underscore the capacity of natural metabolites to maintain mitochondrial homeostasis through multi-target and multi-pathway regulation.

### 4.5 Natural products and mitochondrial energy metabolism in CVD

Cardiomyocytes are among the most energy-demanding cells in the human body and rely heavily on continuous mitochondrial ATP production to sustain normal cardiac function. Maintenance of efficient energy metabolism is therefore essential for cardiac homeostasis, whereas metabolic dysregulation is a common feature of many cardiovascular diseases. Recent studies have demonstrated that a variety of natural products can modulate key steps in energy metabolism and exert important cardioprotective effects.

Ginsenoside Rc, a triterpenoid saponin metabolite isolated from *Panax ginseng* C.A.Mey. (Araliaceae), directly binds to and activates SIRT1, thereby promoting mitochondrial biogenesis and enhancing the expression of respiratory chain complexes, ultimately improving cellular energy metabolism and exerts protective effects in both cardiac and neuronal tissues under ischaemia/reperfusion conditions (Huang et al., 2021). Aloe emodin, an anthraquinone metabolite isolated from *Aloe vera* (L.) Burm. f. (Asphodelaceae), regulates the PTGS2/SH3GLB1/NDP52 axis and stabilises the P4HB emulsification effect on GOT2-mediated kynurenine metabolism, thereby protecting against radiation-induced cardiac injury (Ouyang et al., 2024). Asiatic acid, a pentacyclic triterpenoid metabolite derived from *Centella asiatica* (L.) Urb. (Apiaceae), not only promotes glycogenolysis through activation of the PI3K/Akt pathway to support energy supply, but also activates AMPK-dependent mitophagy. Gene-silencing experiments targeting either AMPK or STBD1 further confirmed the cardioprotective role of asiatic acid in ischemic myocardium (Qiu et al., 2022). Metabolomic analyses in doxorubicin-induced cardiomyopathy models further demonstrated that breviscapine restores myocardial energy homeostasis by regulating serotonin-associated glucose uptake and suppressing excessive fatty acid oxidation. These effects not only improve ATP synthesis efficiency but also preserve cardiac metabolic balance (Li et al., 2022). Cinnamaldehyde, a phenylpropanoid metabolite isolated from *Cinnamomum cassia* (L.) J. Presl (Lauraceae),meanwhile, directly binds to GRK2 and promotes its ubiquitin-mediated degradation, thereby relieving the inhibitory effect of GRK2 on AMPK signalling and enhancing fatty acid oxidative metabolism. Through this mechanism, cinnamaldehyde effectively reverses isoproterenol (ISO)-induced heart failure in rats (Xu et al., 2024).

Gentiopicroside, an iridoid glycoside metabolite derived from *Gentiana scabra* Bunge (Gentianaceae), an iridoid glycoside metabolite derived from *Gentiana scabra* Bunge (Gentianaceae), directly binds to PAQR3, promotes DDB2-mediated ubiquitination and degradation of PAQR3, and thereby relieves PAQR3-dependent inhibition of the PI3K/AKT axis, restoring insulin signalling as well as glucose–lipid metabolic homeostasis (Xiao et al., 2022). Hydroxysafflor yellow A, a chalcone glycoside metabolite derived from *Carthamus tinctorius* L. (Asteraceae), activates malate dehydrogenase 1 (MDH1) through covalent modification of Cys137, enhances MDH1 bioenergetic enzyme activity, restores mitochondrial energy metabolism balance, and improves myocardial ischaemia/reperfusion injury (Zhao et al., 2025).

In summary, these natural products regulate key signalling pathways, modulate enzymatic activities, and reshape the cardiac energy metabolism network, thereby alleviating the energetic crisis associated with heart disease. These findings highlight the potential of natural products as metabolic regulators and provide promising strategies for the development of cardiovascular therapeutics centred on “energy metabolism reprogramming”.

Current evidence suggests that different classes of natural metabolites exhibit distinct regulatory preferences toward mitochondrial function in cardiovascular diseases. Flavonoids, such as baicalin, quercetin, kaempferol, and EGCG, mainly regulate oxidative stress, mitophagy, and inflammatory responses through Nrf2-related pathways and mitochondrial quality control. In contrast, alkaloids represented by berberine and berbamine show stronger effects on AMPK-mediated stress adaptation and energy metabolism, with berberine notably displaying context-dependent bidirectional regulation of autophagy. Saponins, particularly ginsenosides, appear to preferentially regulate mitochondrial biogenesis, mitochondrial dynamics, and respiratory chain function, suggesting more direct effects on mitochondrial bioenergetics and structural remodeling. Meanwhile, polyphenolic metabolites such as curcumin, salvianolic acid B, and tetrahydrocurcumin generally exhibit broader multi-target regulatory properties by simultaneously modulating oxidative stress, inflammation, mitochondrial biogenesis, and cell death pathways. However, these mechanistic classifications remain somewhat simplified, as substantial crosstalk exists among mitochondrial signalling pathways. Future studies should therefore focus more on integrated mitochondrial network regulation rather than isolated pathway interpretation.

### 4.6 Natural product combinations and mitochondrial pathways for CVD treatment

**FIGURE 4:**
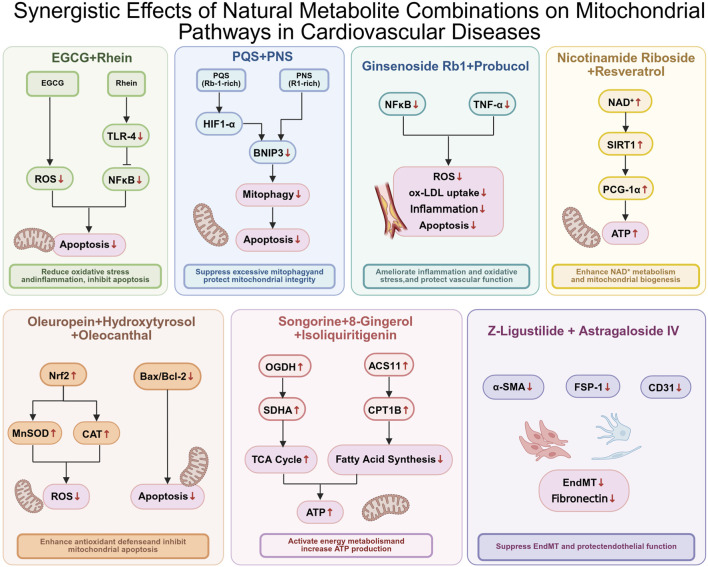
Synergistic effects of natural product combinations in the treatment of cardiovascular diseases through mitochondrial regulation.

With the increasing understanding of the complex pathophysiological mechanisms underlying cardiovascular diseases, single-target therapeutic strategies have revealed notable limitations. In recent years, therapeutic strategies combining natural products have shown distinct benefits. By leveraging the synergistic actions of multiple metabolites that simultaneously target diverse pathways, these approaches modulate mitochondrial function, optimise myocardial metabolism, inhibit fibrotic processes, and alleviate oxidative stress. Collectively, they provide an integrated and multifaceted strategy for cardiovascular disease management. The process by which natural product combinations ameliorate cardiovascular diseases via mitochondrial pathways and related pathways is shown in Table 2 and Figure 4.

**TABLE 2:** Effects of combination of natural products on CVD.

Metabolite name	Function	Animal or cell models	Composition ratio or dosage	Pathway	References
epigallocatechin-3-gallate and rhein	Anti-inflammatory Antioxidant effects	I/R (C57BL/6J:LAD; H9c2,NRCMs: H/R)	Cell: Rh 80 μM+ EGCG 9.81 μM	ROS; TLR4/NF-κB	Liao et al. (2022)
Astragaloside IV derivative (LS102) with Z-ligustilide	Inhibit EndMT	MI (C57BL/6J:LAD; CMECs:TGF-β1)	Astragaloside IV derivative (LS102): Z-ligustilide = 1:1 Animal:LS-102 2 mg/kg + LIG 2 mg/kg Cell:LS-102 5 μM+LIG 5 μM	TGF-β1/Smad Wnt/β-catenin	Liang et al. (2025)
ginsenoside Rb1 and probucol	Anti-inflammatory Antioxidant effects	AS (ApoE^−/−^:HFD; RAW264.7 LPS + IFN-γ)	GRb1:Probucol = 1:1 Animal: Rb1 5 mg/kg + Probucol 5 mg/kg Cell: Rb1 10 μM + Probucol 10 μM	NF-κB/TNF-α; ROS	Zeng et al. (2025)
nicotinamide riboside (NR) and resveratrol (RES)	Improve energy metabolism	I/R (C57BL/6:LAD)	NR:RES = 1:3 Animal: NR:100 mg/kg+ RES:300 mg/kg	NAD^+^	Nie et al. (2021)
Panax quinquefolius saponins and Panax notoginseng saponins	Inhibit excessive mitophagy	I/R (H9c2:H/R)	PQS:PNS = 1:1 Cell:PQS 80 μg/mL+ PNS 80 μg/mL	HIF-1α/BNIP3	Xia et al. (2024)
oleuropein, hydroxytyrosol, oleocanthal	Antioxidant effects Inhibition of mitochondrial apoptosis	MS+IR (C57BL/6:LAD)	OL: HT: OC = 20.6 : 5.9 : 11.6 Animal: Oleuropein (OL): 20.6 mg/kg+ Hydroxytyrosol (HT): 5.9 mg/kg+ Oleocanthal (OC): 11.6 mg/kg	Nrf2	Christodoulou et al. (2024)
Astragaloside IV and Z-ligustilide	Anti-inflammatory Antioxidant effects	MI(C57BL/6J:ISO; HUVECs, HMEC-1: Ang II)	AIV:LIG = 1:2 Animal:20 mg/kg,80 mg/kg Cell: 50 μg/mL	TNF-α, IL-6	Liang et al. (2023)
songorine, 8-gingerol, and isoliquiri-tigenin	Promote the TCA cycle enhance fatty acid β-oxidation	DCM (C57BL/6J DOX H9c2: MDOX)	S:G:I = 3:2:3 Animal: 8 mg/kg/day Cell: 4 μg/mL	Idh2; Ogdh; Sucla2; Sdha, Sdhb,Sdhd; Cpt1b/Cpt2,Acsl1	Ding et al. (2023)

The synergistic mechanisms of natural product combinations in cardiovascular diseases (CVDs) primarily function through the regulation of mitochondrial function and mitochondrial-related signaling pathways.

In the formulation of such combination therapies, the interactions between individual metabolites play a decisive role in shaping therapeutic outcomes. For instance, combined treatment with epigallocatechin gallate (EGCG) and rhein in murine myocardial ischemia/reperfusion models demonstrated complementary mechanisms, in which EGCG effectively scavenged excessive ROS, while rhein suppressed Toll-like receptor 4 (TLR4) signalling. Through simultaneous attenuation of oxidative stress and inflammatory responses, this combination markedly improved ischemic myocardial injury. (Liao et al., 2022). The combined application of the Astragaloside IV derivative LS102 and Z-ligustilide attenuates collagen deposition by suppressing genes involved in extracellular matrix–receptor interactions and cell adhesion pathways. Through these coordinated effects, this combination offers a promising therapeutic approach for the treatment of myocardial fibrosis. (Liang et al., 2025). Ginsenoside Rb1 combined with probucol also exhibited complementary pharmacological properties, in which the glycoside moiety of Rb1 complemented the hydrophobic scaffold of probucol. Following encapsulation into macrophage-derived microvesicles (MMVs), the combination targeted vascular lesions through the VLA-4/ICAM-1 axis, eliminates intracellular ROS, inhibits pro-inflammatory cytokine secretion, and prevents intracellular lipid deposition, thereby effectively delaying atherosclerotic plaque formation (Zeng et al., 2025). Moreover, combined administration of nicotinamide riboside and resveratrol enhanced mitochondrial energy metabolism through coordinated regulation of NAD+ biosynthesis. Specifically, nicotinamide riboside served as an NAD+ precursor, whereas resveratrol activated NMNAT-mediated NAD+ synthesis, collectively increasing the NAD+/NADH ratio and providing protection against cardiac ischemia/reperfusion injury. (Nie et al., 2021). Ligustrazine and its derivative liguzinediol both suppress the Bax/Bcl-2 pathway and inhibit LC3-II conversion, thereby attenuating apoptosis through dual blockade of excessive autophagy and ultimately alleviating pathological cardiac remodeling in doxorubicin-induced heart failure. Importantly, liguzinediol enhances myocardial contractility while avoiding the pro-arrhythmic effects commonly associated with traditional inotropic agents, such as cardiac glycosides. (Lian et al., 2023). Importantly, the synergistic effect of PQS and PNS appears to arise from functional complementarity within distinct mitochondrial regulatory modules. PQS, enriched in ginsenoside Rb1, primarily preserved mitochondrial membrane potential, whereas PNS, enriched in notoginsenoside R1, predominantly regulated HIF-1α signalling. Together, the combination suppressed excessive mitophagy through the HIF-1α/BNIP3 pathway, reduced ROS accumulation, and restored mitochondrial membrane potential (ΔΨm) (Xia et al., 2024).

Optimising the proportions within metabolite combinations is essential to fully realise their synergistic potential. For example, when oleuropein, hydroxytyrosol, and oleocanthal are administered in determined ratios, they not only reduce hyperglycaemia and suppress apoptosis, but also strengthen antioxidant defenses and limit the development of atherosclerotic plaques, thereby providing comprehensive cardiovascular protection. (Christodoulou et al., 2024). Likewise, astragaloside IV combined with Z-ligustilide at a 1:2 ratio markedly attenuated myocardial fibrosis both *in vitro* and *in vivo* by suppressing the TGF-β1/Smad3 pathway, inhibiting endothelial-to-mesenchymal transition (EndMT), and restoring cardiac function. However, the relatively high concentration used *in vitro* (200 μg/mL) raises concerns regarding the physiologically achievable effective concentration after *in vivo* metabolism (Liang et al., 2023). In the case of the SiNi decoction, the combination of three active metabolites, songorine, 8-gingerol, and isoliquiritigenin (at a 3:2:3 ratio), Multi-omics analyses demonstrated that the combination significantly restored the tricarboxylic acid (TCA) cycle and fatty acid oxidation while repairing mitochondrial cristae structure, thereby improving diabetic cardiomyopathy through regulation of mitochondrial energy metabolism (Ding et al., 2023).

Overall, combination strategies involving natural metabolites often exhibit synergistic therapeutic effects in cardiovascular diseases that surpass those achieved by individual metabolites alone. Such synergistic effects arise not only from mechanistic complementarity and coordinated multi-target regulation, but are also highly dependent on dosing strategy, administration timing, and metabolite ratios. Several studies have shown that different ratios may lead to distinct biological outcomes. For example, the active metabolite combination in SiNi decoction achieved superior restoration of mitochondrial energy metabolism at a 3:2:3 ratio, highlighting the importance of rational compatibility design for optimising therapeutic efficacy while potentially reducing dose-related adverse effects.

However, substantial challenges remain regarding the translational applicability of these combinations. Different metabolites often exhibit marked differences in absorption, tissue distribution, metabolic half-life, and elimination pathways, which may result in asynchronous *in vivo* exposure and partially explain why some combinations demonstrate promising efficacy *in vitro* but fail to reproduce comparable therapeutic benefits in animal models. In addition, some concentrations used in current cell experiments may not be physiologically achievable after *in vivo* absorption and metabolism. Therefore, future studies should place greater emphasis on dose optimisation, temporal intervention windows, and the integration of pharmacokinetic and pharmacodynamic evaluation to establish clinically feasible multi-component formulations.

## 5 Discussion

This review comprehensively summarises recent advances in both individual natural metabolites and their rational combinations for the treatment of cardiovascular diseases (CVDs) through the regulation of myocardial mitochondrial function. Increasing evidence suggests that mitochondrial dysfunction, including impaired mitochondrial biogenesis, excessive oxidative stress, dysregulated mitophagy, abnormal mitochondrial dynamics, and metabolic disorders, plays a central role in the pathogenesis of CVDs. Notably, many natural metabolites exert cardioprotective effects through coordinated multi-target regulation of these interconnected mitochondrial processes. For example, berberine simultaneously promotes mitochondrial biogenesis through the SIRT6–AMPK–FOXO3a axis, enhances PINK1/Parkin-mediated mitophagy (Zhou et al., 2024), and suppresses mitochondrial oxidative stress via Nrf2 activation (Wang et al., 2023). Similarly, ginsenoside Rh1 coordinately regulates mitochondrial fusion, fission, and mitophagy to preserve mitochondrial homeostasis under pathological conditions. (Gong et al., 2025). These findings indicate that natural metabolites rarely act through a single pathway, but instead influence multiple mitochondrial functional modules simultaneously, highlighting their unique advantages in complex cardiovascular disorders.

Natural metabolite combinations further demonstrate synergistic and complementary therapeutic effects through coordinated regulation of distinct mitochondrial pathways. For instance, nicotinamide riboside increases myocardial NAD^+^ levels to correct metabolic disturbances, whereas resveratrol promotes NAD^+^ regeneration through NMNAT activation; together, they markedly improve mitochondrial energy metabolism during myocardial ischaemia/reperfusion injury (Nie et al., 2021). Likewise, the optimised 3:2:3 ratio of songorine, 8-gingerol, and isoliquiritigenin in SiNi decoction significantly restores mitochondrial energy homeostasis, (Ding et al., 2023). Suggesting that rational ratio design is essential for maximising therapeutic efficacy. From a pharmacodynamic perspective, combination therapy is far more than a simple additive effect of individual metabolites; rather, it involves coordinated modulation of disease-associated pathways through mechanistic complementarity and precise optimisation of metabolite compatibility. Compared with conventional synthetic drugs that often target single pathways, natural metabolites generally exhibit broader multitarget regulation, lower systemic toxicity, and better biocompatibility, making them promising candidates for mitochondrial-based cardiovascular intervention.

Despite these promising findings, several major challenges continue to hinder clinical translation. Most current evidence remains limited to *in vitro* studies or small-animal models, such as H9c2 cells and rodent primary cardiomyocytes, which cannot fully reproduce the metabolic and electrophysiological properties of human cardiomyocytes. More clinically relevant models, including induced pluripotent stem cell-derived cardiomyocytes (iPSC-CMs), cardiac organoids, and large-animal models, are therefore needed. In addition, substantial differences in the pharmacokinetic properties of natural metabolites may lead to inconsistent *in vivo* exposure, limiting the translational potential of combination therapies. Future studies should therefore place greater emphasis on mitochondria-targeted delivery systems, pharmacokinetic optimisation, and long-term biosafety evaluation. Although natural metabolites are generally considered safer than conventional synthetic drugs, certain metabolites may still induce gastrointestinal discomfort, hepatic metabolic interactions, or dose-dependent toxicity during prolonged administration. Moreover, variability in botanical sources, extraction procedures, and metabolite composition may further affect both efficacy and safety. Importantly, mitochondrial regulation itself is highly context-dependent and bidirectional, suggesting that future studies should focus more on dynamic mitochondrial homeostasis under specific pathological conditions rather than isolated pathway modulation. At present, only limited natural metabolite-based interventions have progressed into clinical cardiovascular studies, and robust evidence from large-scale randomised clinical trials remains insufficient. Therefore, standardisation, clinical validation, and translational studies integrating pharmacokinetics and mitochondrial-targeted strategies will be essential for the future development of natural metabolite-based therapies for cardiovascular diseases.

## 6 Conclusion

In conclusion, natural metabolites and their rational combination strategies exert significant cardioprotective effects through coordinated regulation of multiple mitochondrial functional modules, thereby providing a comprehensive mitochondria-targeted therapeutic strategy for cardiovascular diseases. Compared with conventional synthetic drugs that primarily act on single molecular targets, natural metabolites generally exhibit multi-target, multi-pathway, and bidirectional regulatory properties, together with relatively low systemic toxicity and favorable biocompatibility.

Importantly, rational combination strategies may further enhance therapeutic efficacy through complementary pharmacological actions while simultaneously reducing dose-related adverse effects. Nevertheless, several challenges still limit their clinical translation, including the lack of efficient mitochondria-targeted delivery systems, insufficient *in vivo* pharmacokinetic evaluation, and limited clinical evidence. Future studies integrating multi-omics technologies, systems pharmacology, and artificial intelligence-assisted analysis may facilitate the optimisation of metabolite combination strategies and accelerate their clinical application in cardiovascular diseases.

## References

[B1] Abdullaev I. Gayibov U. Omonturdiev S. Fotima S. Gayibova S. Aripov T. (2025). Molecular pathways in cardiovascular disease under hypoxia: mechanisms, biomarkers, and therapeutic targets. J. Biomed. Res. 39, 254. 10.7555/JBR.38.20240387 40122680 PMC12239985

[B2] Abudureyimu M. Yu W. Cao R. Y. Zhang Y. Liu H. Zheng H. (2020). Berberine promotes cardiac function by upregulating PINK1/parkin-mediated mitophagy in heart failure. Front. Physiol. 11, 565751. 10.3389/fphys.2020.565751 33101051 PMC7546405

[B3] Abudureyimu M. Yang M. Wang X. Luo X. Ge J. Peng H. (2023). Berberine alleviates myocardial diastolic dysfunction by modulating Drp1-mediated mitochondrial fission and Ca2+ homeostasis in a murine model of HFpEF. Front. Med. 17, 1219–1235. 10.1007/s11684-023-0983-0 37656418

[B4] Ajoolabady A. Pratico D. Dunn W. B. Lip G. Y. H. Ren J. (2024). Metabolomics: implication in cardiovascular research and diseases. Obes. Rev. 25, e13825. 10.1111/obr.13825 39370721

[B5] Andreadou I. Daiber A. Baxter G. F. Brizzi M. F. Di Lisa F. Kaludercic N. (2021). Influence of cardiometabolic comorbidities on myocardial function, infarction, and cardioprotection: role of cardiac redox signaling. Free Radic. Biol. Med. 166, 33–52. 10.1016/j.freeradbiomed.2021.02.012 33588049

[B6] Babaei-Kouchaki S. Babapour V. Panahi N. Badalzadeh R. (2020). Effect of troxerutin on oxidative stress and expression of genes regulating mitochondrial biogenesis in doxorubicin-induced myocardial injury in rats. Schmiedeb. Arch. Pharmacol. 393, 1187–1195. 10.1007/s00210-020-01818-0 31960154

[B7] Boeing T. Lívero F. A. Reis Lívero F. A. D. de Souza P. de Almeida D. A. T. Donadel G. (2023). Natural products as modulators of mitochondrial dysfunctions associated with cardiovascular diseases: advances and opportunities. J. Med. FOOD 26, 279–298. 10.1089/jmf.2022.0022 37186894

[B8] Bonora M. Wieckowski M. R. Sinclair D. A. Kroemer G. Pinton P. Galluzzi L. (2019). Targeting mitochondria for cardiovascular disorders: therapeutic potential and obstacles. Nat. Rev. Cardiol. 16, 33–55. 10.1038/s41569-018-0074-0 30177752 PMC6349394

[B9] Bulló M. Papandreou C. García-Gavilán J. Ruiz-Canela M. Li J. Guasch-Ferré M. (2021). Tricarboxylic acid cycle related-metabolites and risk of atrial fibrillation and heart failure. Metabolism 125, 154915. 10.1016/j.metabol.2021.154915 34678258 PMC9206868

[B10] Chambers J. M. Wingert R. A. (2020). PGC-1α in disease: recent renal insights into a versatile metabolic regulator. Cells 9, 2234. 10.3390/cells9102234 33022986 PMC7601329

[B11] Chang X. Zhang Q. Huang Y. Liu J. Wang Y. Guan X. (2024). Quercetin inhibits necroptosis in cardiomyocytes after ischemia–reperfusion via DNA-PKcs-SIRT5-orchestrated mitochondrial quality control. Phytother. Res. 38, 2496–2517. 10.1002/ptr.8177 38447978

[B12] Chen Z. Liang W. Hu J. Zhu Z. Feng J. Ma Y. (2022). Sirt6 deficiency contributes to mitochondrial fission and oxidative damage in podocytes via ROCK1-Drp1 signalling pathway. Cell Prolif. 55, e13296. 10.1111/cpr.13296 35842903 PMC9528772

[B13] Chen L. Lv Y. Wu H. Wang Y. Xu Z. Liu G. (2024). Gastrodin exerts perioperative myocardial protection by improving mitophagy through the PINK1/parkin pathway to reduce myocardial ischemia-reperfusion injury. Phytomedicine 133, 155900. 10.1016/j.phymed.2024.155900 39094441

[B14] Chong B. Jayabaskaran J. Jauhari S. M. Chan S. P. Goh R. Kueh M. T. W. (2024). Global burden of cardiovascular diseases: projections from 2025 to 2050. Eur. J. Prev. Cardiol. 32(11):1001–1015. 10.1093/eurjpc/zwae281 39270739

[B15] Christodoulou A. Nikolaou P.-E. Symeonidi L. Katogiannis K. Pechlivani L. Nikou T. (2024). Cardioprotective potential of oleuropein, hydroxytyrosol, oleocanthal and their combination: unravelling complementary effects on acute myocardial infarction and metabolic syndrome. Redox Biol. 76, 103311. 10.1016/j.redox.2024.103311 39153251 PMC11378258

[B16] Ding L. Li S. Wang F. Xu J. Li S. Wang B. (2021). Berberine improves dietary-induced cardiac remodeling by upregulating kruppel-like factor 4-dependent mitochondrial function. Biol. Chem. 402, 795–803. 10.1515/hsz-2020-0267 33544461

[B17] Ding M. Shi R. Cheng S. Li M. De D. Liu C. (2022a). Mfn2-mediated mitochondrial fusion alleviates doxorubicin-induced cardiotoxicity with enhancing its anticancer activity through metabolic switch. Redox Biol. 52, 102311. 10.1016/j.redox.2022.102311 35413642 PMC9006862

[B18] Ding M. Shi R. Fu F. Li M. De D. Du Y. (2022b). Paeonol protects against doxorubicin-induced cardiotoxicity by promoting Mfn2-mediated mitochondrial fusion through activating the PKCε-Stat3 pathway. J. Adv. Res. 47, 151–162. 10.1016/j.jare.2022.07.002 35842187 PMC10173194

[B19] Ding X. Zhang Y. Pan P. Long C. Zhang X. Zhuo L. (2023). Multiple mitochondria-targeted components screened from sini decoction improved cardiac energetics and mitochondrial dysfunction to attenuate doxorubicin-induced cardiomyopathy. Theranostics 13, 510–530. 10.7150/thno.80066 36632225 PMC9830424

[B20] Gao J. Hou T. (2023). Cardiovascular disease treatment using traditional Chinese medicine:mitochondria as the achilles’ heel. Biomed. and Pharmacother. 164, 114999. 10.1016/j.biopha.2023.114999 37311280

[B21] Gong S. Chen H. Fang S. Li M. Hu J. Li Y. (2025). Ginsenoside Rh1 mitigates mitochondrial dysfunction induced by myocardial ischaemia through its novel role as a sirtuin 3 activator. Br. J Pharmacol. 182, 3017–3035. 10.1111/bph.70022 40151030

[B22] Gutierrez-Mariscal F. M. de la Cruz-Ares S. Torres-Peña J. D. Alcalá-Diaz J. F. Yubero-Serrano E. M. López-Miranda J. (2021). Coenzyme Q10 and cardiovascular diseases. Antioxidants (Basel) 10, 906. 10.3390/antiox10060906 34205085 PMC8229886

[B23] He H. Wang L. Qiao Y. Yang B. Yin D. He M. (2021). Epigallocatechin-3-gallate pretreatment alleviates doxorubicin-induced ferroptosis and cardiotoxicity by upregulating AMPKα2 and activating adaptive autophagy. Redox Biol. 48, 102185. 10.1016/j.redox.2021.102185 34775319 PMC8600154

[B24] Hinton A. Claypool S. M. Neikirk K. Senoo N. Wanjalla C. N. Kirabo A. (2024). Mitochondrial structure and function in human heart failure. Circ. Res. 135, 372–396. 10.1161/CIRCRESAHA.124.323800 38963864 PMC11225798

[B25] Hou D. Liao H. Hao S. Liu R. Huang H. Duan C. (2024). Curcumin simultaneously improves mitochondrial dynamics and myocardial cell bioenergy after sepsis via the SIRT1-DRP1/PGC-1α pathway. Heliyon 10, e28501. 10.1016/j.heliyon.2024.e28501 38586339 PMC10998060

[B26] Hu F. Hu T. Qiao Y. Huang H. Zhang Z. Huang W. (2024). Berberine inhibits excessive autophagy and protects myocardium against ischemia/reperfusion injury via the RhoE/AMPK pathway. Int. J. Mol. Med. 53, 49. 10.3892/ijmm.2024.5373 38577949 PMC10999226

[B27] Huang Q. Su H. Qi B. Wang Y. Yan K. Wang X. (2021). A SIRT1 activator, ginsenoside rc, promotes energy metabolism in cardiomyocytes and neurons. J. Am. Chem. Soc. 143, 1416–1427. 10.1021/jacs.0c10836 33439015

[B28] Huang C. Liu Z. Chen M. Zhang H. Mo R. Chen R. (2024). Up-regulation of BRD4 contributes to gestational diabetes mellitus-induced cardiac hypertrophy in offspring by promoting mitochondria dysfunction in sex-independent manner. Biochem. Pharmacol. 226, 116387. 10.1016/j.bcp.2024.116387 38944397

[B29] Iriondo M. N. Etxaniz A. Varela Y. R. Ballesteros U. Hervás J. H. Montes L. R. (2022). LC3 subfamily in cardiolipin-mediated mitophagy: a comparison of the LC3A, LC3B and LC3C homologs. Autophagy 18, 2985–3003. 10.1080/15548627.2022.2062111 35414338 PMC9673933

[B30] Jiang L. Yin X. Chen Y.-H. Chen Y. Jiang W. Zheng H. (2021). Proteomic analysis reveals ginsenoside Rb1 attenuates myocardial ischemia/reperfusion injury through inhibiting ROS production from mitochondrial complex I. Theranostics 11, 1703–1720. 10.7150/thno.43895 33408776 PMC7778584

[B31] Kumar R. A. Thome T. Sharaf O. M. Ryan T. E. Arnaoutakis G. J. Jeng E. I. (2022). Reversible thiol oxidation increases mitochondrial electron transport complex enzyme activity but not respiration in cardiomyocytes from patients with end-stage heart failure. Cells 11, 2292. 10.3390/cells11152292 35892589 PMC9330889

[B32] Li Y. Feng Y.-F. Liu X.-T. Li Y.-C. Zhu H.-M. Sun M.-R. (2021a). Songorine promotes cardiac mitochondrial biogenesis via Nrf2 induction during sepsis. Redox Biol. 38, 101771. 10.1016/j.redox.2020.101771 33189984 PMC7674615

[B33] Li Y. Wei X. Liu S. Zhao Y. Jin S. Yang X. (2021b). Salidroside protects cardiac function in mice with diabetic cardiomyopathy via activation of mitochondrial biogenesis and SIRT3. Phytotherapy Res. 35, 4579–4591. 10.1002/ptr.7175 34056772

[B34] Li M.-J. Sun W.-S. Yuan Y. Zhang Y.-K. Lu Q. Gao Y.-Z. (2022). Breviscapine remodels myocardial glucose and lipid metabolism by regulating serotonin to alleviate doxorubicin-induced cardiotoxicity. Front. Pharmacol. 13, 930835. 10.3389/fphar.2022.930835 36238546 PMC9551275

[B35] Li G. Pan B. Liu L. Xu X. Zhao W. Mou Q. (2024). Epigallocatechin-3-gallate restores mitochondrial homeostasis impairment by inhibiting HDAC1-mediated NRF1 histone deacetylation in cardiac hypertrophy. Mol. Cell. Biochem. 479, 963–973. 10.1007/s11010-023-04768-2 37266748

[B36] Lian N. Tong J. Zhu W. Meng Q. Jiang M. Bian M. (2023). Ligustrazine and liguzinediol protect against doxorubicin‐induced cardiomyocytes injury by inhibiting mitochondrial apoptosis and autophagy. Clin. Exp. Pharma Physio 50, 867–877. 10.1111/1440-1681.13811 37574718

[B37] Liang P. Bi T. Zhou Y. Ma Y. Liu X. Ren W. (2023). Insights into the mechanism of supramolecular self-assembly in the astragalus membranaceus–angelica sinensis codecoction. ACS Appl. Mater Interfaces 15, 47939–47954. 10.1021/acsami.3c09494 37791782 PMC10591233

[B38] Liang P. Zhang M. Wang Y. Ye T. Wu L. Guo J. (2025). Biomimetic co-delivery of astragaloside IV derivative and Z-ligustilide for targeted inhibition of EndMT in post-myocardial infarction fibrosis. Chem. Eng. J. 523, 168337. 10.1016/j.cej.2025.168337

[B39] Liao X. Song X. Li J. Li L. Fan X. Qin Q. (2022). An injectable co-assembled hydrogel blocks reactive oxygen species and inflammation cycle resisting myocardial ischemia-reperfusion injury. Acta Biomater. 149, 82–95. 10.1016/j.actbio.2022.06.039 35777549

[B40] Lin X. Fei M.-Z. Huang A.-X. Yang L. Ze-Jie Z. Wen G. (2024). Breviscapine protects against pathological cardiac hypertrophy by targeting FOXO3a-mitofusin-1 mediated mitochondrial fusion. Free Radic. Biol. Med. 212, 477–492. 10.1016/j.freeradbiomed.2024.01.007 38190924

[B41] Liu C. Han Y. Gu X. Li M. Du Y. Feng N. (2021). Paeonol promotes Opa1-mediated mitochondrial fusion via activating the CK2α-Stat3 pathway in diabetic cardiomyopathy. Redox Biol. 46, 102098. 10.1016/j.redox.2021.102098 34418601 PMC8385203

[B42] Liu Y. Li M. Du X. Huang Z. Quan N. (2021). Sestrin 2, a potential star of antioxidant stress in cardiovascular diseases. Free Radic. Biol. Med. 163, 56–68. 10.1016/j.freeradbiomed.2020.11.015 33310138

[B43] Liu H. Wang S. Wang J. Guo X. Song Y. Fu K. (2025). Energy metabolism in health and diseases. Signal Transduct. Target Ther. 10, 69. 10.1038/s41392-025-02141-x 39966374 PMC11836267

[B44] Manolis A. S. Manolis A. A. Manolis T. A. Apostolaki N. E. Apostolopoulos E. J. Melita H. (2021). Mitochondrial dysfunction in cardiovascular disease: current status of translational research/clinical and therapeutic implications. Med. Res. Rev. 41, 275–313. 10.1002/med.21732 32959403

[B45] Narendra D. P. Youle R. J. (2024). The role of PINK1-parkin in mitochondrial quality control. Nat. Cell Biol. 26, 1639–1651. 10.1038/s41556-024-01513-9 39358449

[B46] Naumenko N. Mutikainen M. Holappa L. Ruas J. L. Tuomainen T. Tavi P. (2022). PGC-1α deficiency reveals sex-specific links between cardiac energy metabolism and EC-coupling during development of heart failure in mice. Cardiovasc Res. 118, 1520–1534. 10.1093/cvr/cvab188 34086875 PMC9074965

[B47] Nie H. Zhang Y. Yu H. Xiao H. Li T. Yang Q. (2021). Oral delivery of carrier-free dual-drug nanocrystal self-assembled microspheres improved NAD+ bioavailability and attenuated cardiac ischemia/reperfusion injury in mice. Drug Deliv. 28, 433–444. 10.1080/10717544.2021.1886198 33605178 PMC7899691

[B48] Ouyang F. Li Y. Wang H. Liu X. Tan X. Xie G. (2024). Aloe emodin alleviates radiation‐induced heart disease via blocking P4HB lactylation and mitigating kynurenine metabolic disruption. Adv. Sci. 11, 2406026. 10.1002/advs.202406026 PMC11653682 39494721

[B49] Pan D. Chen P. Zhang H. Zhao Q. Fang W. Ji S. (2025). Mitochondrial quality control: a promising target of traditional Chinese medicine in the treatment of cardiovascular disease. Pharmacol. Res. 215, 107712. 10.1016/j.phrs.2025.107712 40154932

[B50] Peñalva D. A. Monnappa A. K. Natale P. López-Montero I. (2024). Mfn2-dependent fusion pathway of PE-enriched micron-sized vesicles. Proc. Natl. Acad. Sci. U. S. A. 121, e2313609121. 10.1073/pnas.2313609121 39012824 PMC11287154

[B51] Poznyak A. V. Ivanova E. A. Sobenin I. A. Yet S.-F. Orekhov A. N. (2020). The role of mitochondria in cardiovascular diseases. Biology 9, 137. 10.3390/biology9060137 32630516 PMC7344641

[B52] Qiu F. Yuan Y. Luo W. Gong Y. Zhang Z. Liu Z. (2022). Asiatic acid alleviates ischemic myocardial injury in mice by modulating mitophagy- and glycophagy-based energy metabolism. Acta Pharmacol. Sin. 43, 1395–1407. 10.1038/s41401-021-00763-9 34522006 PMC9160258

[B53] Rabinovich-Nikitin I. Blant A. Dhingra R. Kirshenbaum L. A. Czubryt M. P. (2022). NF-κB p65 attenuates cardiomyocyte PGC-1α expression in hypoxia. Cells 11, 2193. 10.3390/cells11142193 35883637 PMC9322255

[B54] Ranjbarvaziri S. Kooiker K. B. Ellenberger M. Fajardo G. Zhao M. Vander Roest A. S. (2021). Altered cardiac energetics and mitochondrial dysfunction in hypertrophic cardiomyopathy. Circulation 144, 1714–1731. 10.1161/CIRCULATIONAHA.121.053575 34672721 PMC8608736

[B55] Rios L. Pokhrel S. Li S.-J. Heo G. Haileselassie B. Mochly-Rosen D. (2023). Targeting an allosteric site in dynamin-related protein 1 to inhibit Fis1-mediated mitochondrial dysfunction. Nat. Commun. 14, 4356. 10.1038/s41467-023-40043-0 37468472 PMC10356917

[B56] Rogov A. G. Goleva T. N. Epremyan K. K. Kireev I. I. Zvyagilskaya R. A. (2021). Propagation of mitochondria-derived reactive oxygen species within the dipodascus magnusii cells. Antioxidants (Basel) 10, 120. 10.3390/antiox10010120 33467672 PMC7830518

[B57] Roth G. A. Mensah G. A. Johnson C. O. Addolorato G. Ammirati E. Baddour L. M. (2020). Global burden of cardiovascular diseases and risk factors, 1990–2019. J. Am. Coll. Cardiol. 76, 2982–3021. 10.1016/j.jacc.2020.11.010 33309175 PMC7755038

[B58] Rubio-Tomás T. Soler-Botija C. Martínez-Estrada O. Villena J. A. (2024). Transcriptional control of cardiac energy metabolism in health and disease: lessons from animal models. Biochem. Pharmacol. 224, 116185. 10.1016/j.bcp.2024.116185 38561091

[B59] Scicchitano M. Carresi C. Nucera S. Ruga S. Maiuolo J. Macrì R. (2021). Icariin protects H9c2 rat cardiomyoblasts from doxorubicin-induced cardiotoxicity: role of caveolin-1 upregulation and enhanced autophagic response. Nutrients 13, 4070. 10.3390/nu13114070 34836326 PMC8623794

[B60] Senoner T. Dichtl W. (2019). Oxidative stress in cardiovascular diseases: still a therapeutic target? Nutrients 11, 2090. 10.3390/nu11092090 31487802 PMC6769522

[B61] Shi X. Li Y. Wang Y. Ding T. Zhang X. Wu N. (2021). Pharmacological postconditioning with sappanone a ameliorates myocardial ischemia reperfusion injury and mitochondrial dysfunction via AMPK-mediated mitochondrial quality control. Toxicol. Appl. Pharmacol. 427, 115668. 10.1016/j.taap.2021.115668 34358556

[B62] Su Z.-Z. Li C.-Q. Wang H.-W. Zheng M.-M. Chen Q.-W. (2023). Inhibition of DRP1-dependent mitochondrial fission by mdivi-1 alleviates atherosclerosis through the modulation of M1 polarization. J. Transl. Med. 21, 427. 10.1186/s12967-023-04270-9 37386574 PMC10311781

[B63] Sulkshane P. Ram J. Thakur A. Reis N. Kleifeld O. Glickman M. H. (2021). Ubiquitination and receptor-mediated mitophagy converge to eliminate oxidation-damaged mitochondria during hypoxia. Redox Biol. 45, 102047. 10.1016/j.redox.2021.102047 34175667 PMC8254004

[B64] Sun X. Zhang Y. Chen X.-F. Tang X. (2023). Acylations in cardiovascular biology and diseases, what’s beyond acetylation. EBioMedicine 87, 104418. 10.1016/j.ebiom.2022.104418 36584593 PMC9808004

[B65] Tian X. Huang Y. Zhang X. Fang R. Feng Y. Zhang W. (2022). Salidroside attenuates myocardial ischemia/reperfusion injury via AMPK-induced suppression of endoplasmic reticulum stress and mitochondrial fission. Toxicol. Appl. Pharmacol. 448, 116093. 10.1016/j.taap.2022.116093 35659894

[B66] Wan S. Cui Z. Wu L. Zhang F. Liu T. Hu J. (2023). Ginsenoside rd promotes omentin secretion in adipose through TBK1-AMPK to improve mitochondrial biogenesis via WNT5A/Ca2+ pathways in heart failure. Redox Biol. 60, 102610. 10.1016/j.redox.2023.102610 36652744 PMC9860421

[B67] Wang Y. Liao J. Luo Y. Li M. Su X. Yu B. (2023). Berberine alleviates doxorubicin-induced myocardial injury and fibrosis by eliminating oxidative stress and mitochondrial damage via promoting nrf-2 pathway activation. Int. J. Mol. Sci. 24, 3257. 10.3390/ijms24043257 36834687 PMC9966753

[B68] Wang Z. Wu C. Yin D. Dou K. (2025). Ferroptosis: mechanism and role in diabetes-related cardiovascular diseases. Cardiovasc. Diabetol. 24, 60. 10.1186/s12933-025-02614-x 39920799 PMC11806630

[B69] Wei X.-H. Chen J. Wu X.-F. Zhang Q. Xia G.-Y. Chu X.-Y. (2025). Salvianolic acid B alleviated myocardial ischemia-reperfusion injury via modulating SIRT3-mediated crosstalk between mitochondrial ROS and NLRP3. Phytomedicine 136, 156260. 10.1016/j.phymed.2024.156260 39579610

[B70] Wu J. Chen H. Qin J. Chen N. Lu S. Jin J. (2021). Baicalin improves cardiac outcome and survival by suppressing Drp1‐mediated mitochondrial fission after cardiac arrest‐induced myocardial damage. Oxid. Med. Cell. Longev. 2021, 8865762. 10.1155/2021/8865762 33603953 PMC7870315

[B71] Xia J. Hu J. Zhang R. Liu W. Zhang H. Wang Z. (2022). Icariin exhibits protective effects on cisplatin-induced cardiotoxicity via ROS-mediated oxidative stress injury *in vivo* and *in vitro* . Phytomedicine 104, 154331. 10.1016/j.phymed.2022.154331 35878553

[B72] Xia J. Chen C. Sun Y. Li S. Li Y. Cheng B.-R. (2024). Panax quinquefolius saponins and panax notoginseng saponins attenuate myocardial hypoxia-reoxygenation injury by reducing excessive mitophagy. Cell Biochem. Biophys. 82, 1179–1191. 10.1007/s12013-024-01267-z 38713401

[B73] Xiao H. Sun X. Lin Z. Yang Y. Zhang M. Xu Z. (2022). Gentiopicroside targets PAQR3 to activate the PI3K/AKT signaling pathway and ameliorate disordered glucose and lipid metabolism. Acta Pharm. Sin. B 12, 2887–2904. 10.1016/j.apsb.2021.12.023 35755276 PMC9214054

[B74] Xie W. Deng L. Zhang X. Huang X. Ding J. Liu W. (2024). Myricetin alleviates silica-mediated lung fibrosis via PPARγ-PGC-1α loop and suppressing mitochondrial senescence in epithelial cells. J. Agric. Food Chem. 72, 27737–27749. 10.1021/acs.jafc.4c04887 39586772

[B75] Xu C. Liu Y. Yang J. Zhai M. Fan Z. Qiao R. (2022). Effects of berbamine against myocardial ischemia/reperfusion injury: activation of the 5’ adenosine monophosphate‐activated protein kinase/nuclear factor erythroid 2‐related factor pathway and changes in the mitochondrial state. Biofactors 48, 651–664. 10.1002/biof.1820 35129229 PMC9305777

[B76] Xu Z. Li M. Lyu D. Xiao H. Li S. Li Z. (2024). Cinnamaldehyde activates AMPK/PGC-1α pathway via targeting GRK2 to ameliorate heart failure. Phytomedicine 133, 155894. 10.1016/j.phymed.2024.155894 39089090

[B77] Yang B. Xu Y. Yu J. Wang Q. Fan Q. Zhao X. (2024). Salidroside pretreatment alleviates ferroptosis induced by myocardial ischemia/reperfusion through mitochondrial superoxide-dependent AMPKα2 activation. Phytomedicine 128, 155365. 10.1016/j.phymed.2024.155365 38552436

[B78] Yang N. Zhang R. Zhang H. Yu Y. Xu Z. (2024). ZIP7 contributes to the pathogenesis of diabetic cardiomyopathy by suppressing mitophagy in mouse hearts. Cardiovasc Diabetol. 23, 399. 10.1186/s12933-024-02499-2 39511569 PMC11545574

[B79] Yu L.-M. Dong X. Xue X.-D. Zhang J. Li Z. Wu H.-J. (2019). Naringenin improves mitochondrial function and reduces cardiac damage following ischemia-reperfusion injury: the role of the AMPK-SIRT3 signaling pathway. Food Funct. 10, 2752–2765. 10.1039/C9FO00001A 31041965

[B80] Yue Z. Zhang Y. Zhang W. Zheng N. Wen J. Ren L. (2024). Kaempferol alleviates myocardial ischemia injury by reducing oxidative stress via the HDAC3-mediated Nrf2 signaling pathway. J. Adv. Res. S2090123224004910, 755–764. 10.1016/j.jare.2024.10.037 PMC12789718 39505146

[B81] Zarei M. Sarihi A. Zamani A. Raoufi S. Karimi S. A. Ramezani-Aliakbari F. (2023). Mitochondrial biogenesis and apoptosis as underlying mechanisms involved in the cardioprotective effects of gallic acid against D-galactose-induced aging. Mol. Biol. Rep. 50, 8005–8014. 10.1007/s11033-023-08670-4 37540458

[B82] Zeng J. Zhang Y. Gao Y. Jia M. Guo Y. Li X. (2025). Biomimetic ginsenoside Rb1 and probucol Co-Assembled nanoparticles for targeted atherosclerosis therapy via inhibition of oxidative stress, inflammation, and lipid deposition. ACS Nano 19, 22968–22987. 10.1021/acsnano.5c02492 40534137

[B83] Zhang B. Yang J. Li X. Zhu H. Sun J. Jiang L. (2023). Tetrahydrocurcumin ameliorates postinfarction cardiac dysfunction and remodeling by inhibiting oxidative stress and preserving mitochondrial function via SIRT3 signaling pathway. Phytomedicine 121, 155127. 10.1016/j.phymed.2023.155127 37812853

[B84] Zhang L. Liu X. Wang J. Li Z. Wang S. Yang W. (2025). Kaempferol protects against doxorubicin-induced myocardial damage by inhibiting mitochondrial ROS-dependent ferroptosis. Redox Rep. 30, 2503130. 10.1080/13510002.2025.2503130 40361284 PMC12082743

[B85] Zhang P. Wu H. Lou H. Zhou J. Hao J. Lin H. (2025). Baicalin attenuates diabetic cardiomyopathy *in vivo* and *in vitro* by inhibiting autophagy and cell death through SENP1/SIRT3 signaling pathway activation. Antioxid. Redox Signal. 42, 53–76. 10.1089/ars.2023.0457 38687336

[B86] Zhao X. Wang Z. Wang L. Jiang T. Dong D. Sun M. (2024). The PINK1/parkin signaling pathway-mediated mitophagy: a forgotten protagonist in myocardial ischemia/reperfusion injury. Pharmacol. Res. 209, 107466. 10.1016/j.phrs.2024.107466 39419133

[B87] Zhao F. Cheng W. Wu D. Gong Z. Ma W. Xu J. (2025). Hydroxysafflor Yellow a Ameliorates Myocardial ischemia/reperfusion Injury by Promoting Mdh1-mediated Mitochondrial Metabolic Homeostasis. 10.2139/ssrn.5102526 40513321

[B88] Zhong C. Xie Y. Wang H. Chen W. Yang Z. Zhang L. (2024). Berberine inhibits NLRP3 inflammasome activation by regulating mTOR/mtROS axis to alleviate diabetic cardiomyopathy. Eur. J. Pharmacol. 964, 176253. 10.1016/j.ejphar.2023.176253 38096968

[B89] Zhou Z. Zhao Q. Huang Y. Meng S. Chen X. Zhang G. (2024). Berberine ameliorates chronic intermittent hypoxia-induced cardiac remodelling by preserving mitochondrial function, role of SIRT6 signalling. J. Cell. Mol. Med. 28, e18407. 10.1111/jcmm.18407 38894630 PMC11187832

[B90] Zuo B. Li X. Xu D. Zhao L. Yang Y. Luan Y. (2024). Targeting mitochondrial transfer: a new horizon in cardiovascular disease treatment. J. Transl. Med. 22, 1160. 10.1186/s12967-024-05979-x 39741312 PMC11687156

